# Single colony genetic analysis of epilithic stream algae of the genus *Chamaesiphon* spp

**DOI:** 10.1007/s10750-017-3295-z

**Published:** 2017-08-21

**Authors:** Rainer Kurmayer, Guntram Christiansen, Andreas Holzinger, Eugen Rott

**Affiliations:** Research Institute for Limnology, University of Innsbruck, Mondseestraße 9, 5310 Mondsee, Austria; Institute of Botany, University of Innsbruck, Sternwartestraße 15, 6020 Innsbruck, Austria

**Keywords:** Gravel streams, Heteropolar cyanobacteria, Bioindication, Single colony PCR, 16S rDNA gene sequencing

## Abstract

In order to understand *Chamaesiphon* spp. evolution and ecological diversification, we investigated the phylogenetic differentiation of three morphospecies from field samples by means of single colony genetics. Individual colonies of three different morphospecies (*C. starmachii, C. polonicus, C. geitleri,*) were isolated from lotic gravel streams and their 16S rDNA nucleotide variability was analyzed. For a number of individual colonies, microscopical and ultrastructural analysis was also performed. A phylogenetic tree of all major lineages of the phylum of Cyanobacteria assigned all *Chamaesiphon* genotypes (1149–1176 bp) most closely with the family of Gomontiellaceae of the order Oscillatoriales. The sequences obtained from colonies assigned to *C. starmachii* (*n* = 21), *C. polonicus* (*n* = 9), and *C. geitleri* (*n* = 17) were found to reveal high average (3.5%) nucleotide diversity. No phylogenetic sub-branching in correspondence with morphology was observed suggesting that the three *Chamaesiphon* morphospecies did not represent monophyletic taxa. We could not attribute specific thylakoid ultrastructure to phylogenetic sub-branches; however, the observed parietally and loosely arranged thylakoids indicate that for the genus *Chamaesiphon*, the variability in thylakoid ultrastructure might have been underestimated. In summary, the high nucleotide diversity of the 16S rDNA gene implies phylogenetic diversity that corresponds little to morphological classification.

## Introduction

The cyanobacterial morphogenus *Chamaesiphon* represents one of the most widespread taxa forming thin biofilms in mountain rivers worldwide (e.g., Bürgi et al., 2003; Rott et al., 2006; Rott, 2008; Sant’Anna et al., 2011; Scott & Marcarelli, 2012; Gutowski et al., 2015). Several *Chamaesiphon* species can cover up to >70% of the wetted perimeter in clear mountain streams (Rott & Wehr, 2016). The genus *Chamaesiphon* was recorded over a broad range of environmental situations in respect to (i) light (i.e., from shaded to highly light (UV) exposed sites), (ii) pH (i.e., from acid to alkaline, Cantonati et al., 2007) and (iii) nutrients (i.e., from ultra-oligotrophic to eutrophic conditions, Rott et al., 1999; Rott & Schneider, 2014). Up to date, 33 species of the morphogenus *Chamaesiphon* have been validly described under the rules of the Botanical Nomenclatoric Code (ICBN, Komárek & Anagnostidis, 1999, and Cyano Database: http://www.cyanodb.cz, February, 2017). These cyanobacterial species are distinguished based on morphological characters, and although some morphological characters are less stable than others, several of the morphospecies of the genus *Chamaesiphon* have been recorded repeatedly from different regions in the world (e.g., Komárek & Anagnostidis, 1999; Rott & Wehr, 2016). Microbiological typification defines the morphogenus *Chamaesiphon* as a simple unicellular cyanobacterium with typical asymmetrical binary fission (called budding or exocyte or exospore formation) produced at one pole of the mother cell, where typically one single or a consecutive large series of small exospores (or small buds) is formed (Herdman et al., 2001).

Colony forming cyanobacteria can reach a macroscopically visible size from clonal cellular growth. This facilitates the isolation of single individuals directly from field samples for analyses of their genetics, cyanotoxins or other bioactive secondary metabolites (e.g., Kurmayer et al., 2002). Advanced molecular techniques have increased the sensitivity of PCR, resulting in the possibility to amplify DNA fragments from just picograms of template DNA, such as from single colonies of cyanobacteria. This makes it possible to perform dozens of PCR experiments amplifying large DNA fragments (>1000 bp) from a single specimen (e.g. Chen et al., 2016). Combining the field-based techniques with advanced molecular biological methods holds great potential to analyze the ecological, as well as phylogenetic, diversification of cyanobacteria, particularly if they are difficult to cultivate (e.g., Mareš & Cantonati, 2016). In general, a detailed molecular resolution of *Chamaesiphon* morphospecies might allow a better characterization of running water habitats that are under environmental stress induced by regional climate change or by anthropogenic influence (e.g., Loza et al., 2013).

The aim of this study was to characterize *Chamaesiphon* morphospecies using molecular biological techniques on the individual (single colony) scale, which would allow to relate morphological descriptors to genotype variability. We speculated that morphological differentiation might correlate with phylogeny, even between morphospecies, because of selective environmental pressure resulting in phylogenetically distinct ecotypes. For example, high mechanical pressure in lotic ecosystems may lead to morphospecies forming a multilayered cell wall and growing in colonies (assigned to *Chamaesiphon* subgenus *Godlewskia*, Komárek & Kaštovský, 2003; Hoffmann et al., 2005) specialized to grow in the viscous sublayer on solid substrates. Thus, the question arises whether it is possible to characterize individual *Chamaesiphon* morphospecies by molecular taxonomic markers such as 16S rDNA genes and/or the more variable internal transcribed spacer regions.

## Materials and methods

### Study organisms and sampling area

**Fig. 1:**
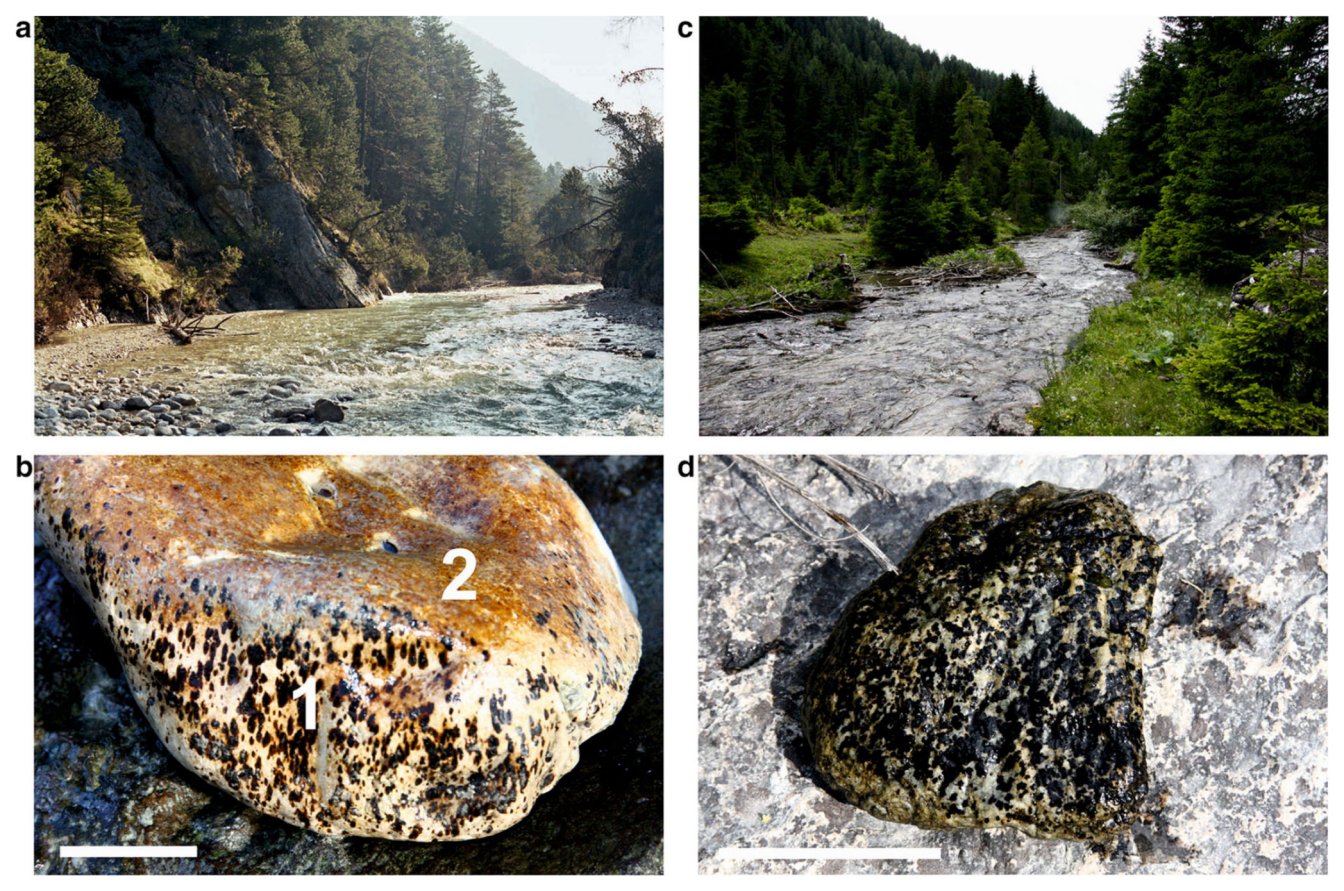
**a** Calcareous river Isar (Tyrol) and **b** pebble from Isar with (*1*) *dark brown spots* formed by *C. geitleri* on the lateral side and (*2*) *red crust* formed by *C. polonicus*, **c** central alpine river Nederbach and **d** pebble from Nederbach with black spots formed by *C. starmachii*. *Scale bars* 5 cm

In alpine gravel streams, epilithic *Chamaesiphon* species were recognized forming small patches (*C. starmachii* Kann 1972, *C. geitleri* Luther 1954) or thin and sometimes extended covers (*C. polonicus* (Rostafinski) Hansgirg 1892), see Fig. 1. The two sampling sites included crystalline geology (Nederbach, Ochsengarten, near Kühtai, Tyrol, Austria) and carbonaceous basement (Isar River, Scharnitz, near Seefeld, Tyrol and Karwendelbach, a tributary of Isar River, see Table 1).

In addition, the following *Chamaesiphon* strains available from culture collections were used (Table 2): *C. minutus* (PCC 6605), *C. subglobosus* (PCC 7430) (Rippka et al., 1979), *C. polonicus* (SAG 32.87), *C. polymorphus* Geitler 1925 (CCALA Hindák 1984/5). Two *Chamaesiphon* morphospecies were isolated following established protocols (Chu, 1942): epilithic growing *C. geitleri* from Gschnitzbach (Trins, Tyrol, 47°04′38.73″N, 11°24′46.46″O, 1191 m), and the epiphytic morphospecies *C.* cf. *incrustans* from a small spring (Nature Reserve Fontanili di Corte Val Rei, near Reggio Emilia, Italy 44°46′ 2″N, 10° 31′ 58″E, 30 m a.s.l.). In order to obtain purified clonal cultures the strains were streaked on agar repeatedly.

### Single *Chamaesiphon* colony isolation

From the sampling sites, individual pebbles overgrown with *Chamaesiphon* were sampled and transported into the laboratory under cold and dark conditions. Individual *Chamaesiphon* colonies (*C. starmachii, C. polonicus, C. geitleri*) were directly selected and isolated from the substratum under the dissecting microscope using a sterile razor blade and a needle. The individual colonies were transferred to an agar plate (2% agarose, Chu, 1942) and purified by moving the colony gently on agar using a fine sterile wire loop and a sterile fine needle. Aliquots of individual colonies were stored in 10 μl Millipore water. One aliquot was used as PCR template and stored frozen (−20°C). Another aliquot of the colony was assigned to morphospecies identification according to Komárek & Anagnostidis (1999). A third aliquot was used for TEM analysis.

### Light- and transmission electron microscopy

Light microscopy was performed using a Zeiss Axiovert 200 M microscope and images were captured with a Zeiss Axiocam Mrc 5 and an Olympus BMX50 microscope with a ProGresCT3 camera, respectively. Transmission electron microscopy was performed as described (Holzinger et al., 2009). Briefly, cells were fixed with 2.5% glutaraldehyde for 1.5 h at room temperature, followed by a fixation in 1% OsO_4_ at 4°C overnight (18 h), both in 50 mM caccodylate buffer (pH 6.8). Samples were dehydrated in increasing ethanol concentrations, embedded in modified Spurr’s resin and heat polymerized at 80°C. Ultrathin sections were prepared and viewed at a Zeiss Libra 120 transmission electron microscope (80 kV) connected to a ProScan 2k SSCCD camera, operated by OSIS iTEM software. Images were processed with Adobe Photoshop elements 11. Cyanobacterial inclusions were assigned according to the recent catalog of cyanobacterial ultrastructure, i.e., the carboxysomes were recognized as electron dense bodies (Gonzalez-Esquer et al., 2016).

### Single *Chamaesiphon* colony PCR

Single colonies were dispersed using ultrasonication and suspended in PCR sample dilution buffer resulting in 50 μl total colony sample volume as described (Chen et al., 2016). The extracted DNA was used directly as a template for PCR amplification of the 16S rDNA and internal transcribed spacer (ITS) region (359F, 23S30R, ca. 1.6 kbp, Taton et al., 2003). In a pilot experiment, a number of colonies were tested for successful PCR amplification using two different PCR DNA-polymerases (i) a classical Taq polymerase (DreamTaq DNA Polymerase, Thermo Fisher Scientific, Darmstadt, Germany) and (ii) the engineered high speed, proof-reading polymerase (Phusion, Thermo Fisher Scientific). The PCR amplifications were performed in a total volume of 25 μl. For the DreamTaq PCR, the reaction mixture included 2.5 μl DreamTaq PCR Buffer (10×, including 20 mM MgCl_2_), 1.0 μl MgCl_2_ (25 mM), 0.75 μl dNTPs (10 mM each), 0.75 μl of each primer (10 μM each), 0.15 μl of DreamTaq polymerase, 16.6 μl sterile Millipore water, and 2.5 μl of template. For the Phusion PCR the reaction mixture included 5 μl of (5×) Phusion GC reaction buffer, 0.5 μl of dNTPs, 1.25 μl of each primer (10 μM), 0.25 μl of Phusion DNA polymerase, 14.25 μl sterile Millipore water and 2.5 μl DNA template. For the DreamTaq/Phusion PCR, the thermal cycling protocol included an initial denaturation step at 94°C (3 min)/98°C (30 s), followed by 35 cycles including denaturation at 94°C (30 s)/98°C (10 s), annealing at 60°C (30 s)/60°C (15 s), and elongation 72°C (1.5 min)/72°C (30 s). During a pilot experiment, the Dream Taq Polymerase had little PCR success and was not used further. Using the Phusion polymerase, we were able to amplify the 16S rDNA from the majority of the isolated colonies from three different morphospecies (Table S1).

### Clone library, RFLP-typing and sequencing

To identify rDNA genotypes of individual *Chamaesiphon* colonies, for each morphospecies the obtained 16S rDNA-ITS PCR products (1.6 kbp) were purified and ligated into the pJet-Cloning Vector system (Thermo Fisher Scientific) and cloned through subsequent transformation of *Escherichia coli* (strain K 12) following manufacturer’s instructions. Purified plasmids were sequenced from both directions using pJet-Cloning Vector standard primers and assembled sequences were submitted to DDBJ/EMBL/Genbank under the accession no. KY704109–KY704162 (Table S2).

In a pilot experiment, for two colony samples of the three *Chamaesiphon* morphospecies (*C. starmachii, C. polonicus*, *C. geitleri*), 20 colonies were randomly selected and dissolved in 10 μl of Millipore water and 1 μl was used as template for PCR amplification using the same primers (see above). Subsequent restriction analysis (AluI) revealed the genetic diversity within the individual *Chamaesiphon* colony samples as well as potentially co-occurring other cyanobacteria. By this method, when comparing the AluI restriction profile between the amplified and sequenced PCR product from strain *C. minutus* PCC 6605 (1798 bp, access no. KY704112) and the three copies of 16S rDNA-ITS-23S rDNA (1798 bp) in the sequenced genome (Shih et al., 2013) identical restriction profiles were obtained (8 substitutions, 99.5% similarity). When comparing different morphospecies from the individual *Chamaesiphon* colonies one to three AluI restriction types were observed (Table S1). In only one case a restriction type (1044 bp) with 99% sequence identity to *Microcoleus vaginatus* SAG 22.11 (EF654074.1) occurred. Since cyanobacteria other than *Chamaesiphon* were amplified and sequenced in a single case only, it was concluded that the single colony isolation technique was reproducible for the genus *Chamaesiphon*, while the influence of other potentially co-occurring cyanobacterial taxa on the PCR results was of minor importance.

### Statistical and phylogenetic analysis

Sequences were aligned and conserved motifs were analyzed according to Iteman et al. (2000). While the 16S rDNA comprised 1149–1176 bp until the highly conserved motif D1 (Table 3), the ITS region was located between the highly conserved motif D1 and the less conserved motif D5 resulting in 608–681 bp. The sequence similarity was calculated using the PHYLIP package (version 3.69, Felsenstein, 2004). All obtained 16S rDNA sequences (1149–1176 bp) were included in a phylogenetic tree constructed from previously described cyanobacterial taxa comprising all major lineages of the phylum (Wilmotte & Herdmann, 2001) as well as clades found related to *Chamaesiphon* more recently (Bohunická et al., 2015; Mareš & Cantonati, 2016). In total, 150 non-redundant sequences obtained from strains were downloaded from the Ribosomal Database Project (RDP), Cole et al., 2014 (Table S3). The 16S rDNA phylogenetic tree was constructed by maximum likelihood method using the program RAxML 8.0.26 (Stamatakis, 2014), executing 1000 rapid bootstrap inferences and thereafter a thorough ML search. Likelihood of the final tree was evaluated and optimized using a Gamma substitution matrix (GTR).

## Results

### Light microscopical characterization of *Chamaesiphon* morphospecies

The three morphospecies sampled from the field had macroscopically visible features as follows: *C. starmachii* and *C. geitleri* were forming dark brown to almost black coatings (thickness 0.1–1 mm) on stony substrates (a few square millimeters were typical for *C. starmachii* and *C. geitleri* in early stages; older colonies of *C. starmachii* also formed extended coatings). In contrast *C. polonicus* colonies did not form thicker delimited patches and were characterized by thin covers or crusts colored orange to dark red-brown (Fig. 1).

**Fig. 2:**
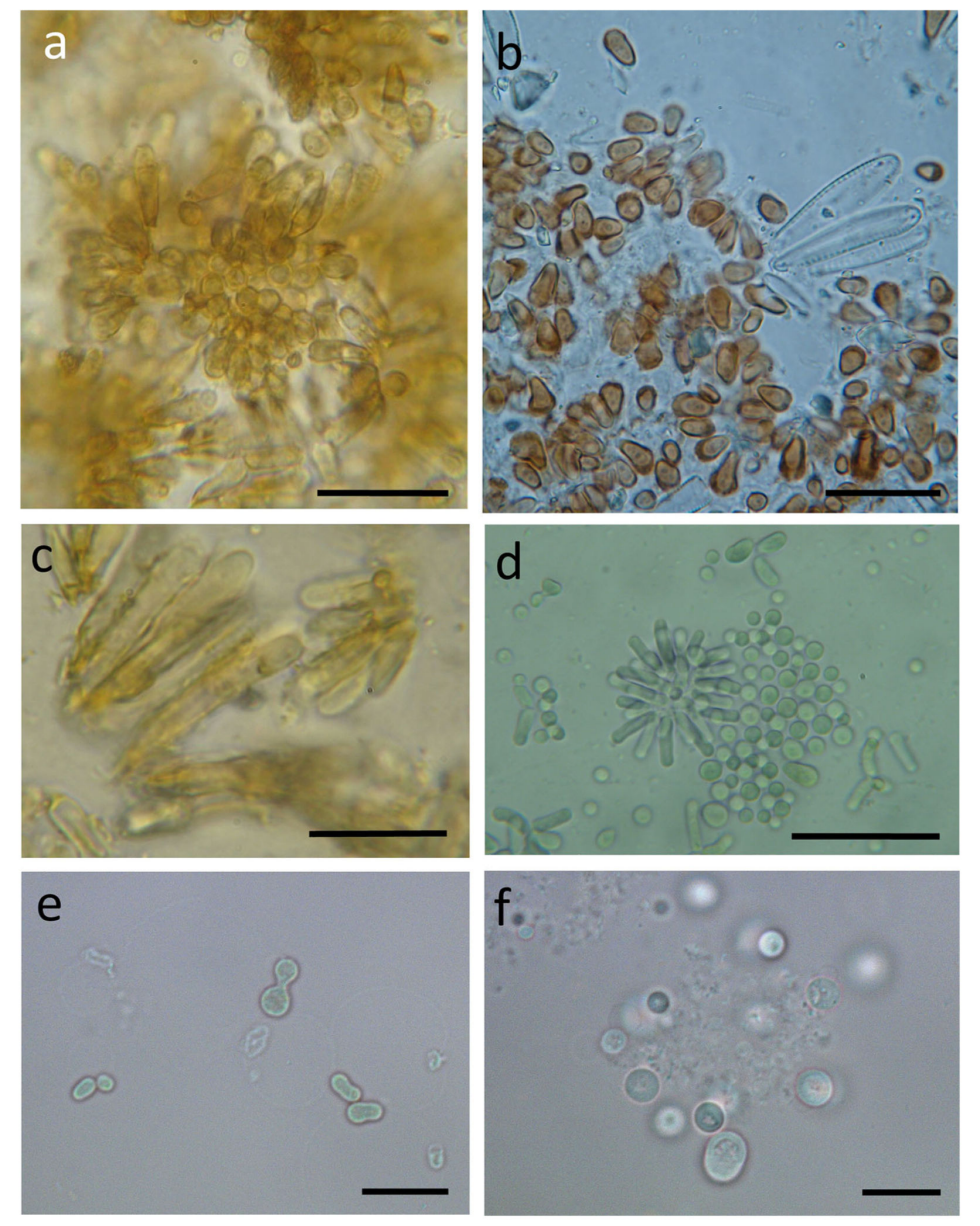
Light micrographs of *Chamaesiphon* spp. **a–c** squash slide samples of field material and **d–f** cultivated strains. **a** *C. starmachii* from Nederbach, **b** *C. polonicus* from River Isar, **c** *C. geitleri* from River Isar, **d** *C.* cf. *incrustans* strain No. 1036, **e** strain PCC 6605 (*C. minutus*), **f** strain PCC 7430 (*C. subglobosus*). *Scale bars*: **a, d** 20 μm, **b**: 15 μm, **c, e, f**: 10 μm

The microscopical aspects of young stages of all three morphospecies were first colorless and similar to each other. However, later in growth typical morphological differences of the three taxa became evident, i.e., a gradual coverage by several specifically pigmented layers of exopolymers (pseudovaginae). *C. starmachii* had an inverse egg-shaped to cylindrical form (Fig. 2a), *C. polonicus* cells were broad spherical sometimes cup-shaped or pear-shaped and formed ellipsoid persisting stages narrowing at the base (Fig. 2b), and *C. geitleri* cells were mostly of elongated elliptic shape with a short inverse egg-shaped basis (Fig. 2c). Exospores (buds) of all three species were primarily spherical but developed differently between the three species. *C. geitleri* pseudosporangia developed into elongated cells (five to eight fold longer than wide), *C. starmachii* pseudosporangia also grew in length (but only three to five fold longer than wide), whereas *C. polonicus* pseudosporangia were only two to three fold longer than wide. Maturing cells of all three morphospecies formed gradually multilayered extracellular sheath coatings (pseudovagina) before the sheath is breaking at the apex thereby releasing the naked exospores. The thickest and most multilayered dark yellow to brown colored pseudovagina was found in *C. geitleri*. The pseudovagina of *C. polonicus* was normally orange to dark red-brown colored. Under desiccated conditions, *C. polonicus* lost its thick pseudovagina layers and the cell shape became regularly elongated and elliptic with striking red pigmentation. The mature cells differed in size between the three taxa (Table S1). Cultivated strains PCC 6605 (*C. minutus*), PCC 7430 (*C. subglobosus*), and strain No.1036 (*C.* cf. *incrustans*) typically lost their sheaths, but kept asymmetric cell division (Fig. 2d–f). The strain culture of *C*. cf. *incrustans* also showed heteropolar cells forming exocytes at one side of the elongated mother cell.

### Ultrastructural characterization of *Chamaesiphon* morphospecies

**Fig. 3:**
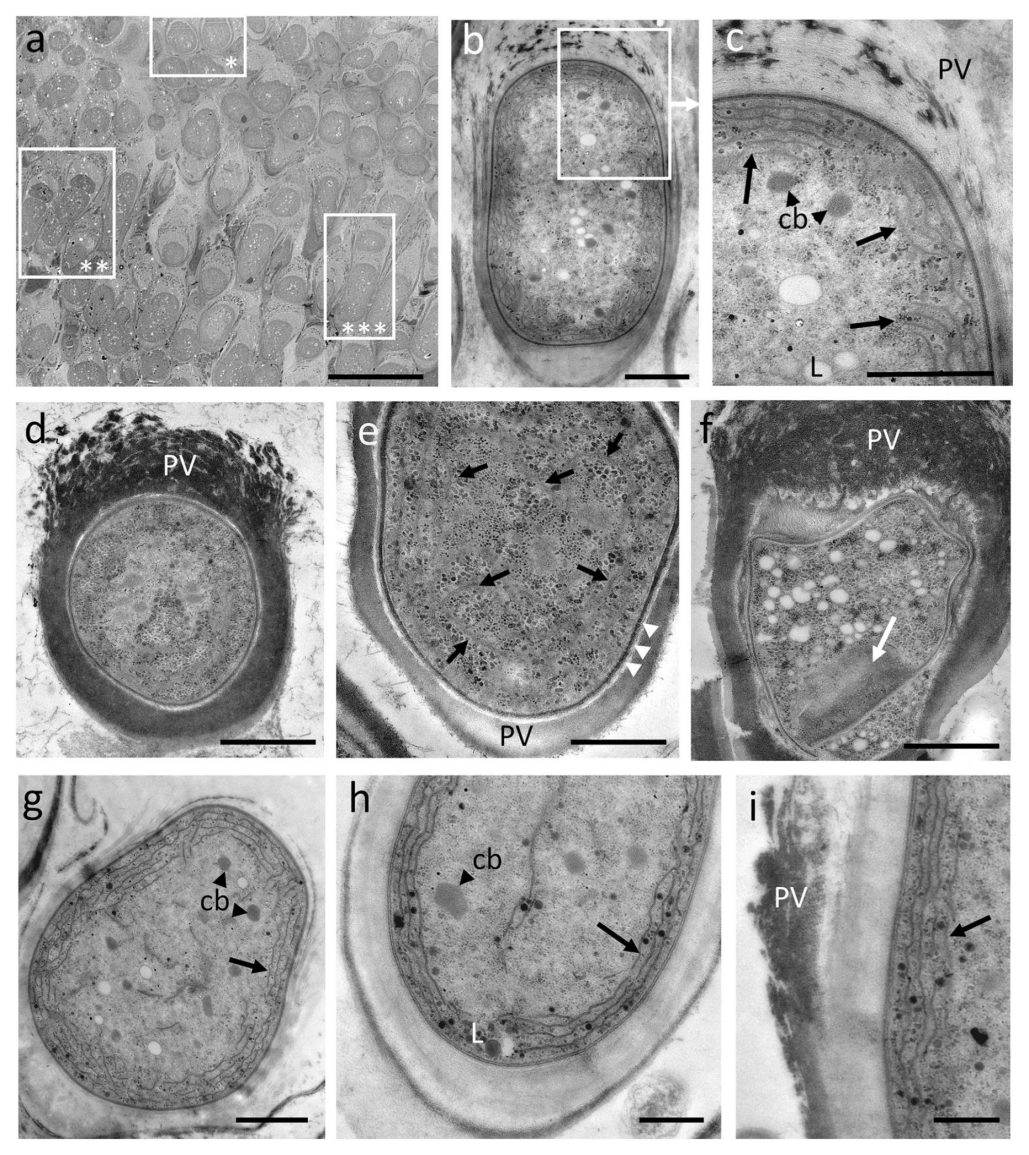
Transmission electron micrographs of field samples of *Chamaesiphon* spp. **a–c** *C. starmachii* from Nederbach: **a** cross section of multilayered cell aggregation; asterisks indicate three successive stages of unequal cell budding, **b** *C. starmachii*, mature pseudosporangium with upward broken pseudovagina, **c** detail of **b** showing several peripheral bundles of thylakoid membranes (*arrows*), **d–f** *C. polonicus* from River Isar: **d** encysted pseudosporangium with electron dense sheath layer partly frayed in the upward part where exospores would be released, **e** viable cell with thylakoid double membranes distributed over the whole cell cross section (*arrows*); white arrowheads indicate mucus secreting pores, **f** cross section of a drought stressed exosporangium with parts of the cell wall exhibiting the many basal mucus pores (white arrow), **g–i** *C. geitleri* from River Isar, notice the wall parallel thylakoids (*arrows*), **g** presumably extending young exospore, **h–i** details of mature exosporangia, **i** wall parallel thylakoids (*arrow*). *cb* carboxisomes, *L* lipid body, *PV* pseudovagina. *Scale bars*: **a** 10 μm, **b**, **g**, **d** 1 μm, **c**, **e–f**, **h–i** 500 nm

The ultrathin cross section of several *C. starmachii* colonies exhibited variations of cell shapes and sizes arranged in multiple layers with older (darker) cells in the basal part of the colony (showing a thick apically frayed or widely open pseudovagina and a conical cell basis), and younger (smaller) cells in the upper layers respectively (Fig. 3a). Three different stages of unequal binary fission/division with perpendicular cross walls and gradual separation of increasing elliptic naked single exospores were observed (as marked by asterisks). The cellular ultrastructure of a mature pseudosporangium of *C. starmachii* showed a mostly peripheral arrangement of a number of non-continuous bundles of thylakoids (4–5–6 irregular broken arrays) (Fig. 3b, c). Within the cytoplasm distinct central areas with medium electron density and no thylakoids were observed (Fig. 3c).

A similar cell architecture was observed for *C. polonicus* (Fig. 3d–f), however, with pores perforating the cell wall enriched in the lower (basal) part of the cell (Fig. 3e, f), which are putatively used for mucilage secretion. Cross sections of *C. polonicus* showed a mature pseudosporangium with a partly frayed thick dark pigmented slime layer in the upper part, before releasing the exospores (Fig. 3d). Double membranes of thylakoids were visible (Fig. 3e). In this case, the irregularly bent thylakoids appeared to cover almost the whole cell cross section.

In *C. geitleri*, there was a distinct arrangement of the thylakoid bundles (mostly 3–4) parallel to the cell wall (Fig. 3g–i). Only few individual thylakoids were also found in the cell center (Fig. 3h). The sheath on the outer side of the cell wall had an irregular appearance (Fig. 3i). In Fig. 3g and h presumably young cells of *C. geitleri* started to grow in length. The lower part of an exosporangium had a thicker but less pigmented mucilage layer as compared with an older cell showing very thick and dark pigmented sheath layers (Fig. 3i). Compared with other morphospecies the number of thylakoids was lower and in some cases additional thylakoids in the cell center were visible (e.g. in Fig. 3h). Further details of the cells constituents included carboxysomes in the cell center, lipid bodies, and glycogen granules (see the abbreviations in Fig. 3).

**Fig. 4:**
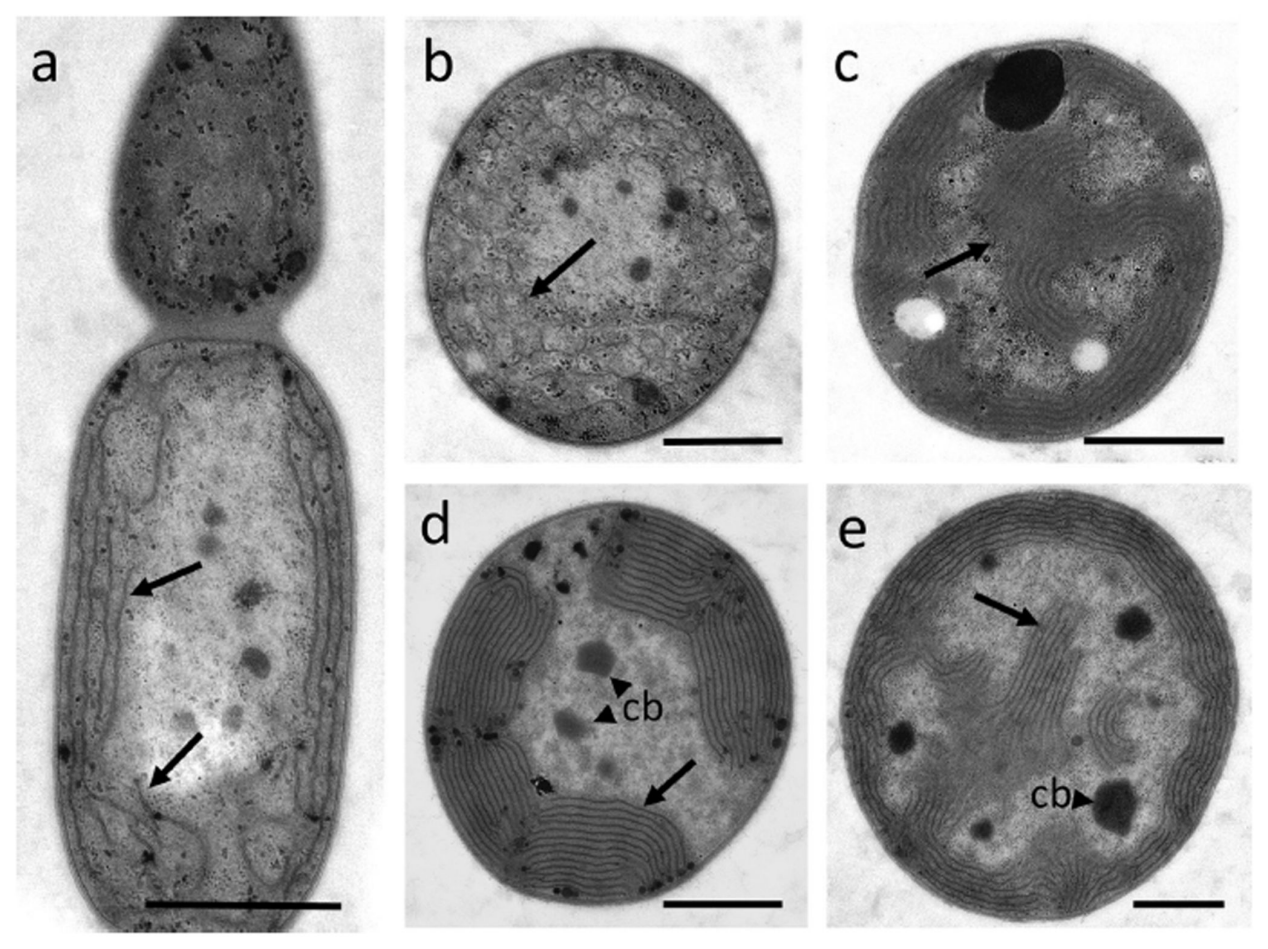
Transmission electron micrographs of different *Chamaesiphon* spp. strains. **a** *C.* cf. *incrustans* No. 1036, (**b**, **c**) PCC 6605 (*C. minutus*), (**d**, **e**) PCC 7430 (*C. subglobosus*). **a** status in cultivation after thin layered pseudovagina had been lost, thylakoids arranged wall parallel (*arrows*), **b** cortical arrays of thylakoids in older cell, **c** tangential section shows thylakoids distributed throughout the cell, **d** thylakoids organized in a few, more or less long, ribbons. **e** tangential section with similar appearance as in **c**, i.e. thylakoids protruding towards the cell center. *Scale bars*: 1 μm

Cells from *Chamaesiphon* strain cultures had a distinct ultrastructure, without additional sheath layers covering the cells. In general, the uneven cell division was maintained under culture conditions (e.g., Figure 4a). In Fig. 4b, the cell constituents were found almost disintegrated. *Chamaesiphon* cf. *incrustans* strain No. 1036 was isolated from a rich epiphytic layer of long mature exosporangia assigned to *C.* cf. *incrustans* with a one-layered transparent pseudovagina. Under culture conditions the cells lost the pseudovagina and showed either thylakoid membranes parallel to the cell wall or a more irregular thylakoid distribution (Fig. 4a). Notably, *C. minutus* (PCC 6605, Fig. 4b, c) and *C. subglobosus* (PCC 7430, Fig. 4d, e) showed large areas with numerous distinct thylakoid membranes parallel to the cell wall. In tangential sections, the thylakoid membranes were distributed over the whole area (Fig. 4c, e).

### Genetic variability within individual *Chamaesiphon* colonies and between morphospecies

In total fifteen individual colonies of *C. starmachii* (resulting in 21 sequences), five individual colonies of *C. polonicus* (9 sequences) and six individual colonies of *C. geitleri* (17 sequences) were genetically analyzed. In general, the variation within each morphospecies was high (on average 2–3.8%, Table 3). Using BLASTn all obtained 16S rDNA sequences (1149–1176 bp) showed highest identity (97–99%) with 16S rDNA of *C. minutus* PCC 6605 (Access. No. CP003600, Shih et al., 2013) or *C. subglobosus* PCC 7430 (access. No. AY170472). The total nucleotide variation was max. 15% and on average 3.5%. The genetic variation within the ITS region was even higher and was not further analyzed (Table S4).

**Fig. 5:**
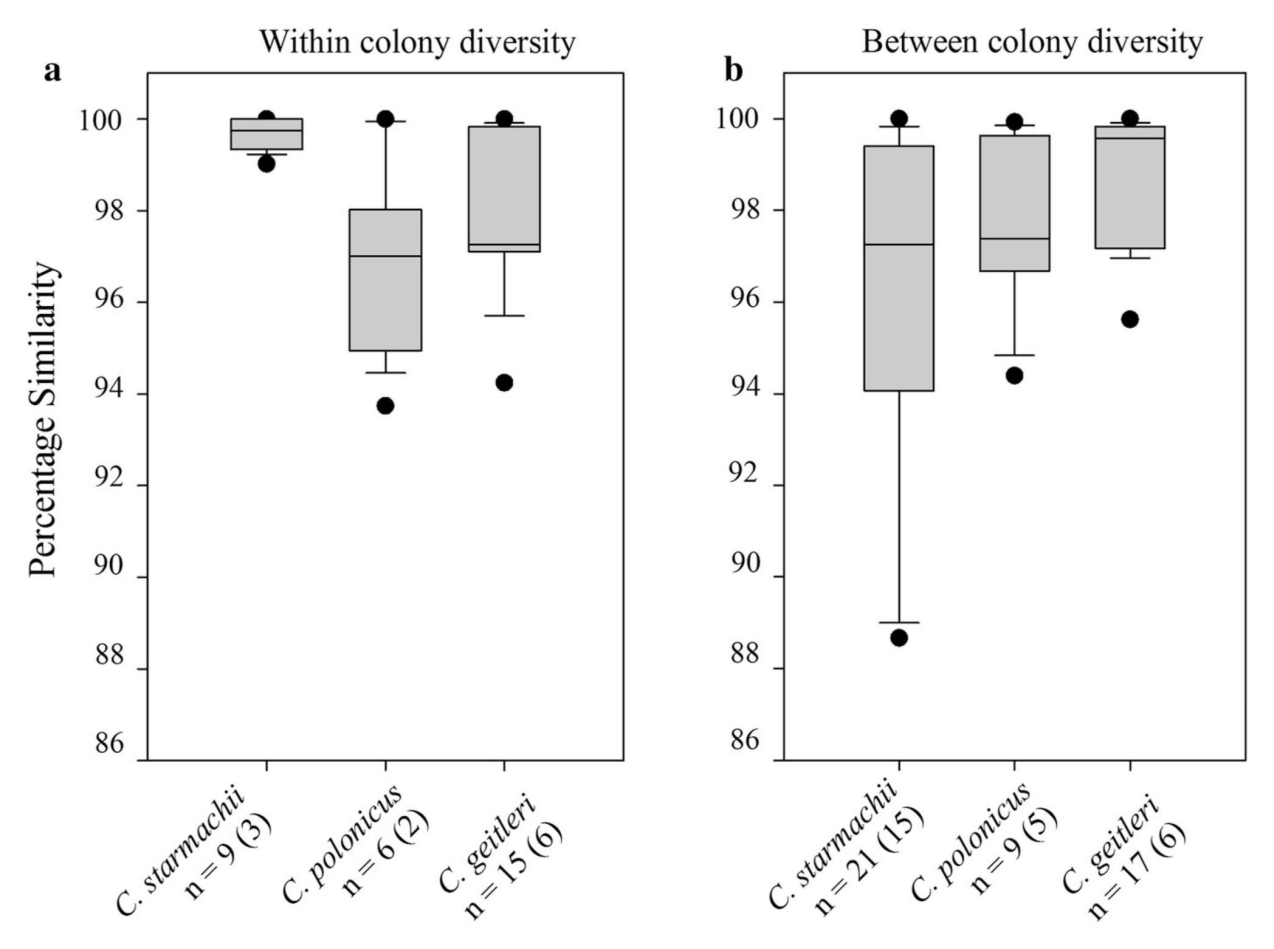
Percentage of similarity in 16S rDNA sequences **a** within individual *Chamaesiphon* colonies and **b** between individual colonies of one specific *Chamaesiphon* morphospecies. *n* = sample sizes of sequences (colonies), (see also Table 3 and Table S5)

In order to estimate the 16S rDNA genetic variation observed within single isolated *Chamaesiphon* colonies, a few clones of PCR products were sequenced and the 16S rDNA nucleotide variability within one isolated colony was recorded. Within individual colonies of *C. starmachii* and *C. geitleri* a relatively low genetic variation of 16S rDNA was found (<1%, Table S5). The sequences were almost identical or showed a few base pair substitutions and/or point deletions, indicating that clonal genotypes were isolated. In contrast, the *C. polonicus* colonies showed higher within-colony genetic variation (0–5%) (Fig. 5a).

Including all sequences obtained from specific morphospecies led to an increase of the genetic diversity for the morphospecies *C. starmachii* (Fig. 5b). Compared with the nucleotide diversity recorded within *C. starmachii* colonies, the increase in nucleotide variability was due to the comparison between all isolated *C. starmachii* colonies (*n* = 15). From the increased nucleotide variation between individual colonies assigned to *C. starmachii*, it is concluded that high genetic diversity occurred within the *C. starmachii* morphospecies.

### Phylogenetic analysis

**Fig. 6:**
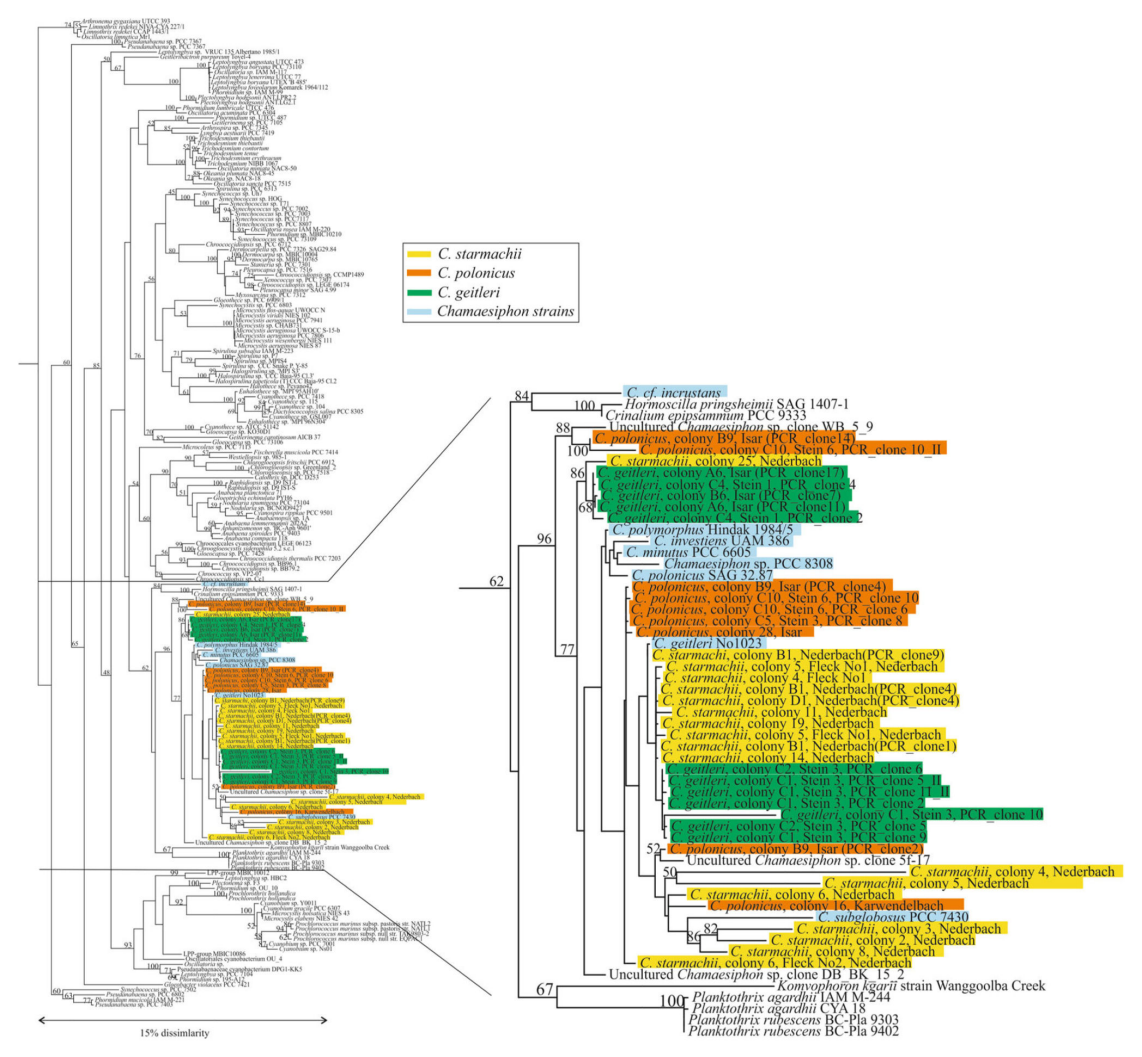
Maximum likelihood phylogenetic tree calculated from 16S rDNA (1121 bp) from *Chamaesiphon* individual colonies and numerous cyanobacteria strains downloaded from the Ribosomal Database Project (RDP). For sequence accession numbers see Table S3. Numbers at nodes indicate the percent bootstrap frequency (1000 replicates) using RAxML (Only bootstrap values of >50% are shown). *Blue Chamaesiphon* strains as listed in Table 2, *yellow C. starmachii*, *orange C. polonicus*, *green C. geitleri*. For sequence accession numbers see Table S3. The ML-tree was rooted using *Escherichia coli*

The phylogenetic maximum likelihood tree comprising 16S rDNA sequences from all major lineages of the phylum suggested that the morphogenus *Chamaesiphon* is most closely related to the family of Gomontiellaceae, which is phylogenetically placed among other groups of filamentous cyanobacteria that are part of the Oscillatoriales (sensu Komárek & Anagnostidis, 2005). All *Chamaesiphon* sequences were found phylogenetically distinct from *Geitleribactron*, a sheath-lacking heteropolar cyanobacterium grouped more closely with the freshwater *Leptolyngbya* lineage (sensu Wilmotte & Herdman, 2001). The same phylogenetic branching was obtained when using the Neighbor-Joining algorithm (data not shown). Notably, the most divergent strain assigned to *C.* cf. *incrustans* (*Chamaesiphon* sensu stricto sensu Komárek & Kaštovský, 2003), which was isolated from a small spring near Reggio Emilia in Italy, was grouped with the Gomontiellaceae represented by *Hormoscilla pringsheimii* strain SAG 1407-1 and *Crinalium epipsammum* strain PCC 9333 (Fig. 6). All other *Chamaesiphon* individual colonies and strains (assigned to subgenus *Chamaesiphon* and *Godlewskia* sensu Komárek & Kaštovský, 2003; Hoffmann et al., 2005) formed a distinct sister clade. However, the individual morphospecies *C. starmachii, C. polonicus*, and *C. geitleri* were found distributed across the entire phylogenetic branch. In this phylogeny, *C. polonicus* and *C. geitleri* were both located within sequences obtained from *C. starmachii*. The high genetic diversity of the *C. starmachii* morphospecies, as observed from genetic variability (Fig. 5), could be inferred from the phylogenetic tree as well. Except of *C.* cf. *incrustans* all other *Chamaesiphon* strains were also found distributed among the sequences obtained from individual *Chamaesiphon* morphospecies. *C. geitleri* strain No. 1023 clustered closely with *C. starmachii* and *C. geitleri* individual colonies isolated from the field. In summary, the results imply that (i) the *Chamaesiphon* morphospecies assigned to subgenera *Chamaesiphon* and *Godlewskia* formed a polyphyletic group, and (ii) the high nucleotide diversity with the 16S rDNA gene did not correlate with morphological diversity.

## Discussion

### Methodology of single colony genetic analysis

Despite its wide ecological distribution, the phylogenetic position of the genus *Chamaesiphon* is relatively unexplored. This is at least partly because rather few strains have been cultivated. Cultivation-independent methods such as the genetic analysis of single colonies of morphospecies can provide an alternative, which has been used successfully in the past (Mareš et al., 2015; Mareš & Cantonati, 2016). During the isolation procedure under the light microscope, associated taxa growing on and between *Chamaesiphon* colonies were observed (e.g., *Homoeothrix varians*), however, not recognized from 16S rDNA sequences obtained from purified *Chamaesiphon* colonies using the cyanobacteria-specific primers from Taton et al. (2003). In addition, small (epiphytic) coccoid cyanobacteria of the genera *Aphanothece* or *Aphanocapsa* were never observed through sequencing. The purity of the isolated colonies was supported by TEM analysis. Finally, the 16S rDNA sequences typically revealed *Chamaesiphon* as “closest relative” after BLASTn analysis. Thus, even if small-celled coccoid cyanobacteria would have been overlooked in the microscope, the BLASTn analysis of 16S rDNA sequences would have shown their possible co-occurrence. From these results, it is concluded that isolation and purification of *Chamaesiphon* colonies on agar plates was sufficiently pure to obtain *Chamaesiphon*-specific results.

In the TEM analysis, the morphospecies of *C. starmachii* and *C. geitleri* revealed mostly irregular delimited thylakoid bundles arranged in a various number of arrays along the periphery of the cells. For *C. polonicus* the irregular arrangement of the thylakoids across the cell content was observed. We suggest that during the growth and aging process a gradual detachment of thylakoids within the peripheral bundles takes place, which is similar to the observations for some Gomontiellaceae (Bohunická et al., 2015). In general, the cultivated *Chamaesiphon* strains, *C.* cf. *incrustans* No. 1036, *C. minutus* PCC 6605 and *C. subglobosus* PCC 7430 showed more regular staples of thylakoids. However, *C. minutus* shows the same variability in thylakoid structure as *C. subglobosus* despite that the latter species should be included into subgenus *Godlewskia*. This variability in thylakoid structure within PCC strains does not conform with the findings reported previously from *C. subglobosus* (PCC 7430, see Table 2C/g in Komárek & Anagnostidis, 1999) and *C. minutus* (PCC 6605 in Gonzalez-Esquer et al., 2016). In summary, it is concluded that the variability in thylakoid ultrastructure might have been underestimated in the genus *Chamaesiphon*.

### *Chamaesiphon* phylogenetic relationship as inferred from 16S rDNA gene loci

Among prokaryotes cyanobacteria are the most morphologically diverse phylum, both in aquatic and terrestrial environments. In general, the phylogeny inferred from 16S rDNA sequences corresponded with the phylogeny inferred from a larger number of gene loci obtained through genomic sequencing of more than hundred strains (e.g., Komárek et al., 2014). The only *Chamaesiphon* species currently with a sequenced genome, *C. minutus* strain PCC 6605, was found most closely related to *Crinalium epipsammum* PCC 9333 and the *Trichodesmium*-*Oscillatoria* PCC 7515 lineage XII (sensu Wilmotte & Herdman, 2001), which is corresponding to the phylogenetic results based on 16S rDNA (Fig. 6). Thus it is very likely that the 16S rDNA results obtained in this study will be corresponding to phylogenetic analysis obtained from sequenced genomes in the future. The high genetic diversity within the 16S rDNA gene found in the morphogenus *Chamaesiphon* possibly suggests a long evolutionary history. Such high diversity (on average 3.5%, max. 15%) is significantly above the recommended value for separation of species (Stackebrandt & Goebel, 1994) and has forced reclassification of existing genera into several independent genera, most prominently for the genus *Synechococcus* (e.g., Robertson et al., 2001). In contrast to *Synechococcus*, however, *Chamaesiphon* shows a much higher morphological diversity (e.g., Komárek & Anagnostidis, 1999), which does not correspond to 16S rDNA genotyping. Furthermore, the three *Chamaesiphon* morphospecies analyzed by single colony genetics were found distributed over one clade, which could be assigned to two or even more lineages with bootstrap support. The 16S rDNA genes of the morphospecies from the *Chamaesiphon* strains were found intermixed with those of the single colony morphospecies with only one exception (*C.* cf. *incrustans*). This phenomenon would suggest that the current *Chamaesiphon* subgenus classification system (*Chamaesiphon s. stricto*, *Chamaesiphonopsis*, and *Godlewskia* (Komárek & Anagnostidis, 1999) is indeed polyphyletic.

Relaxed molecular clock analyses suggested that morphological traits related to the formation of microbial mats (e.g., larger cell diameters, filamentous growth, sheath) were not visible from microfossils until the diversification of the three major lineages after 2.3 billion years (Blank & Sanchez-Baracaldo, 2010). The same authors propose that the first large-diameter filamentous microfossils to appear in the rock record at 1.9 billion years (according to Golubic & Seong-Joo, 1999) were early members of the PNT clade (containing *Pseudanabaena*, *Nostocales* and *Trichodesmium*), and probably also included the early ancestors of *Chamaesiphon*. Epibiotic coccoid microfossils from Gaoyuzhuang Formation, Hebei Province, northern China dated 1.4 billion years ago were described to show polarized growth and cyanobacterial sheaths (Seong-Joo et al., 1999). The authors further stated that the reproductive mode of fossil epibionts corresponded to the pattern of asymmetric transverse division or budding characteristic of the extant *Chamaesiphon* genus. The authors emphasized large and diversified populations and the occurrence of widespread epibiosis. Thus, it seems possible that the morphological differentiation of *Chamaesiphon* is an old evolutionary character, which possibly evolved in response to competition for space in shallow stromatolithic-like environments.

### Niche diversification of *Chamaesiphon* morphospecies

It is generally accepted that in unstable river beds, such as gravel pit streams, benthic cyanobacteria can persist because thick sheaths protect the cells from mechanical stress and drying (Kann, 1978). In analogy to rocky subaerial habitats, cyanobacteria can also tolerate fast-flowing and fluctuating aquatic environments by forming multiple cell wall layers of a sticky extracellular matrix, which also protect the cells from UV, drying and mechanical abrasion (Helm & Potts, 2012). Thus the formation of multilayers in *Chamaesiphon* might also be understood as a response to mechanical stresses that occur in lotic habitats. Notably, a number of *Chamaesiphon* morphospecies in stream biofilms have been currently used in water quality assessment and bioindication (Schneider & Lindström, 2011; Rott & Schneider, 2014; Rott & Wehr, 2016). This niche diversification has been attributed to both different geochemical preferences (e.g., *C. fuscus* (Rostafinski) Hansgirg 1888 occurs in low carbonate waters vs. *C. geitleri* occurs in high carbonated waters, whereas *C. polonicus* showed no geochemical preference) as well as to eutrophication (*C. polymorphus* is considered a eutraphentic taxon). It is not known whether specific morphospecies cannot occur under specific conditions due to physiological constrains (i.e., desiccation, excess UV radiation, shear stress) or if a particular species is competitively overgrown by a better adapted morphospecies. In either case, this ecophysiological adaptation could not be resolved by 16S rDNA sequencing since closely related individual genotypes showed substantial overlap among the observed morphospecies. In other words, the most closely related 16S rDNA genotypes assigned to *C. starmachii* and *C. geitleri* occurred both in low carbonate vs. high carbonate water, respectively. The high variability in 16S rDNA among *Chamaesiphon* morphospecies, which is not corresponding with observed ecological niches, also restricts the use of 16S rDNA genotyping for bioindication. Thus the genetic variability of the 16S ribosomal gene does not allow the recognition of the three investigated morphospecies. The three morphospecies have been validly described under the ICBN based on morphological characters (Komárek & Anagnostidis, 1999), but using the modern polyphasic approach would need to be re-evaluated, i.e., clonal cultures of individual morphospecies will need to be analyzed for exopolymer production and sheath biochemical composition, accessory carotenoid and sunscreening pigment composition, as well as ecophysiological characters such as desiccation ability. For example, special UV shielding pigments and specific pigment (phycocanin/phycoerythrin) composition were recently studied and differences found between *C. starmachii*/*C. geitleri* and *C. polonicus* (Siegfried Aigner, personnel communication). This conclusion is of relevance because DNA barcoding techniques are intensively discussed as a high throughput tool to characterize environmental communities. For example, diatom barcodes are used as bioindicators in the European Water Framework Directive (Rimet et al., 2016). In this study, individual *Chamaesiphon* morphospecies could not be recognized by means of 16S rDNA, restricting the use of 16S rDNA in bioassessments. We think that for a general evaluation of metabarcoding approaches, the approach used in this study (i.e. identification of cyanobacteria colonies and filaments in the microscope according to morphological criteria and then isolating and analyzing by PCR based methods for marker genes) could be indeed useful.

## Supplementary Material

**Electronic supplementary material** The online version of this article (doi:10.1007/s10750-017-3295-z) contains supplementary material, which is available to authorized users.

Supplemental Information

## Figures and Tables

**Table 1:** Sampling sites and observed *Chamaesiphon* spp. morphospecies

Site	Coordinates	Altitude (m. a. SL.)	Parameters (Cond.^a^, Temp, pH)	*Chamaesiphon* morphospecies	Sampling Date
Nederbach (Ochsengarten or Zirmbach)	47°13′45.16″N	1593	46 µS, 10°C, pH 7.3	*C. starmachii*	Nov’09, Apr’10, Apr’11
	10°57′28.98″O				
Isar (Scharnitz, Isarursprung)	47°23′01.86″N	980	200 µS, 5°C, pH 8	*C. polonicus*, *C. geitleri*,	Apr’10, Apr’11, Oct’15
	11°16′20.49″O				
Karwendelbach (Scharnitz, Isarursprung)	47°22′49.53″N	984	800 µS, 9°C, pH 8	*C. polonicus*	Apr’10
	11°17′5.41″O				

a Conductivity (µS cm^−1^)

**Table 2:** Clonal cultures of *Chamaesiphon* spp.

Strain name	Date isolated	Origin	Morphospecies
CCALA HINDAK 1984/5	1984	Lake Millstätter See (AT)	*C. polymorphus*
PCC 6605	1966	Stream, Berkeley, California (USA)	*C. minutus*
PCC 7430	1963	Stream, Sarka Valley near Prague (CR)	*C. subglobosus*
PCC 8308	1983	Epiphyte on *Cladophora* sp., from Lake Vierwaldstättersee, Kastanienbaum (CH)	*Chamaesiphon sp.*
SAG 32.87	1965	Stansstad, moist rocks (CH)	*C. polonicus*
No. 1036	2012	Epiphyte on *Cladophora* in a spring pool (IT)	*C.* cf. *incrustans*
No. 1022, 1023, 1024	2014	Gschnitzbach, near Trins, Tirol (AT)	*C. geitleri*

*CCALA* Culture collection of autotrophic organisms Institute of Botany CAS, Trebon, *PCC* Pasteur culture collection, *SAG* Sammlung Algenkulturen Göttingen

**Table 3:** Summary of 16S rDNA variability from *Chamaesiphon* strain cultures and individually isolated *Chamaesiphon* morphospecies colonies

*Chamaesiphon* species	Length (bp) min–med–max	Max. dissimilarity (%)	Average similarity (%)	*N*
*C. starmachii*	1158–1168–1176	14.7	96.2	21 (15)
*C. polonicus*	1166–1168–1173	6.3	97.5	9 (5)
*C. geitleri*	1149–1167–1169	6.0	98	17 (6)
*Chamaesiphon* spp. (strains)	1162–1167–1169	9.6	95.6	7 (7)
Total	1149–1168–1176	15.0	96.5	54

*N* Number of sequences (individual colonies)
