# Supplementary material for: Single colony genetic analysis of epilithic stream algae of the genus Chamaesiphon spp
Source: Hydrobiologia. Author manuscript; Available in PMC 2018 Apr 1. (PMC5856356; doi:10.1007/s10750-017-3295-z)
Supplement: Supplemental Information [file NIHMS76059-supplement-Supplemental_Information.docx]

**Supplemental Information**

Table S1. Results on the PCR amplification of the rDNA 16S-23S region using the primers from Taton et al. (2003) from *Chamaesiphon* colonies isolated in the microscope and assigned to three different morphospecies.

|  | *C. geitleri* | *C. polonicus* | *C. starmachii* |
| --- | --- | --- | --- |
| Cell dimensions (µm), (length × width (without sheath),  (n = 10 for each morphospecies) | 13.1 × 2.7 (2.1) | 6.3 × 4 (3) | 9.2 × 4.4 (4) |
| Colony with PCR product  (n = 8 for each morphospecies) | 5 | 5 | 6 |
| AluI restriction types in 20 clones per colony | 1 to 3 | 3 | 1 to 3 |
| Genotypes phylogenetically assigned to *Chamaesiphon* | 2 to 4 | 3 | 3 to 6 |
| Genotypes phylogenetically not assigned to *Chamaesiphon* | 0 | 0 | 1 |

Table S2. Partial 16S-ITS rDNA sequences submitted to NCBI and access. No. (shading indicates sequences obtained from various PCR clones from the same colony of one *Chamaesiphon* morphospecies)

| Date of isolation | Description | Sequ_name | Access. No. | Length (bp) |
| --- | --- | --- | --- | --- |
| 22. Nov 09 | *C. starmachii*, individ. Colony 2, Nederbach, v. Agar | >2_st_NB | KY704116 | 1811 |
| 22. Nov 09 | *C. starmachii*, individ. Colony 3, Nederbach, v. Agar | >3_st_NB | KY704117 | 1802 |
| 22. Nov 09 | *C. starmachii*, individ. Colony 4, Nederbach, v. Agar | >4_st_NB | KY704118 | 1813 |
| 22. Nov 09 | *C. starmachii*, individ. Colony 5, Nederbach, v. Agar | >5_st_NB | KY7041196 | 1786 |
| 22. Nov 09 | *C. starmachii*, individ. Colony 6, Nederbach, v. Agar | >6_st_NB | KY704120 | 1810 |
| 22. Nov 09 | *C. starmachii*, individ. Colony 8, Nederbach, v. Agar | >Ch8_sp_ZB | KY704121 | 1803 |
| 11. Apr 10 | *C. starmachii*, indiv. Col. 19, Nederbach, u. Mikroskop isoliert | >10_st_NB | KY704122 | 1807 |
| 11. Apr 10 | *C. starmachii*, indiv. Col. 25, Nederbach, u. Mikroskop isoliert | >13_st_NB | KY704123 | 1769 |
| 11. Apr 10 | *C. starmachii*, indiv. Col. 28, Isar, u. Mikroskop isoliert | >1_po_IS | KY704124 | 1830 |
| 21.06.2010 | *C. starmachii*, indiv. Col. 4, Fleck No. 1, Nederbach, u. Mikroskop isoliert | >14_st_NB | KY704125 | 1808 |
| 21.06.2010 | *C. starmachii*, indiv. Col. 5, Fleck No. 1, Nederbach, u. Mikroskop isoliert, (PCR_clone B1) | >15_st_NB | KY704126 | 1808 |
| 21.06.2010 | *C. starmachii*, indiv. Col. 5, Fleck No. 1, Nederbach, u. Mikroskop isoliert, (PCR_clone B9) | >16_st_NB | KY704127 | 1808 |
| 21.06.2010 | *C. starmachii*, indiv. Col. 6, Fleck No. 2, Nederbach, u. Mikroskop isoliert | >17_st_NB | KY704128 | 1823 |
| 23.03.2010 | *C. starmachii*, individ. Colony 11, Nederbach, v. Agar | >18_st_NB | KY704129 | 1807 |
| 23.03.2010 | *C. starmachii*, individ. Colony 14, Nederbach, v. Agar | >19_st_NB | KY704130 | 1805 |
| 17.02.2010 | *C. polonicus*, indiv. Col. 16, Karwendelbach, v. Agar | >2_po_KW | KY704131 | 1794 |
| 26.04.2011 | *C. starmachii,* indiv. colony B1, Nederbach(PCR_clone1) | >20_st_NB | KY704132 | 1806 |
| 26.04.2011 | *C. starmachii*, indiv. colony B1, Nederbach(PCR_clone3) | >21_st_NB | KY704133 | 1806 |
| 26.04.2011 | *C. starmachii*, indiv. colony B1, Nederbach(PCR_clone4) | >22_st_NB | KY704134 | 1805 |
| 26.04.2011 | *C. starmachii*, indiv. Colony B1, Nederbach(PCR_clone9) | >23_st_NB | KY704135 | 1811 |
| 26.04.2011 | *C. starmachii* indiv. Col. D1, Nederbach(PCR_clone2) | >24_st_NB | KY704136 | 1806 |
| 26.04.2011 | *C. starmachii*, indiv. Col. D1, Nederbach(PCR_clone4) | >25_st_NB | KY704137 | 1806 |
| 26.04.2011 | *C. starmachii*, indiv. Col. D1, Nederbach(PCR_clone7) | >26_st_NB | KY704138 | 1806 |
| 26.04.2011 | *C. geitleri*, indiv. Col. A6, Isar (PCR_clone11) | >1_ge_IS | KY704139 | 1824 |
| 26.04.2011 | *C. geitleri*, indiv. Col. A6, Isar (PCR_clone17) | >2_ge_IS | KY704140 | 1827 |
| 26.04.2011 | *C. geitleri*, indiv. Col. B6, Isar (PCR_clone1) | >3_ge_IS | KY704141 | 1824 |
| 26.04.2011 | *C. geitleri*, indiv. Col. B6, Isar (PCR_clone17) | >4_ge_IS | KY704142 | 1824 |
| 26.04.2011 | *C. geitleri*, indiv. Col. B6, Isar (PCR_clone9) | >5_ge_IS | KY704143 | 1827 |
| 26.04.2011 | *C. geitleri*, indiv. Col. B6, Isar (PCR_clone7) | >6_ge_IS | KY704144 | 1826 |
| 26.04.2011 | *C. polonicus*, indiv. Col. B9, Isar (PCR_clone14) | >3_po_IS | KY704145 | 1793 |
| 26.04.2011 | *C. polonicus*, indiv. Col. B9, Isar (PCR_clone2) | >4_po_IS | KY704146 | 1809 |
| 26.04.2011 | *C. polonicus*, indiv. Col. B9, Isar (PCR_clone4) | >5_po_IS | KY704147 | 1832 |
| 05.07.2011 | *C.* *polymorphus* Hindak 1984/5 | >pl_1984 | KY704110 | 1845 |
| 16.04.2013 | *C. polonicus* SAG 32.87 | >po_32_87 | KY704111 | 1807 |
| 01.07.2010 | *C. minutus* PCC 6605 | >min_6605 | KY704112 | 1798 |
| 01.07.2010 | *C. subglobosus* PCC 7430 | >sub_7430 | KY704113 | 1798 |
| 01.07.2010 | *C.* sp. PCC 8308 | >sp_8308 | KY704114 | 1786 |
| 16.04.2013 | *C.* cf. *incrustans* strain No. 1036 | >incr_VR | KY704115 | 1769 |
| 22.10.2015 | *C. geitleri*, indiv. Col., C1, Pebble 3, PCR_clone 2 | >7_ge_IS | KY704148 | 1802 |
| 22.10.2015 | *C. geitleri*, indiv. Col., C1, Pebble 3, PCR_clone 9 | >8_ge_IS | KY704149 | 1802 |
| 22.10.2015 | *C. geitleri*, indiv. Col., C1, Pebble 3, PCR_clone 10 | >9_ge_IS | KY704150 | 1799 |
| 22.10.2015 | *C. geitleri*, indiv. Col., C2, Pebble 3, PCR_clone 5 | >10_ge_IS | KY704151 | 1803 |
| 22.10.2015 | *C. geitleri*, indiv. Col., C2, Pebble 3, PCR_clone 6 | >11_ge_IS | KY704152 | 1802 |
| 22.10.2015 | *C. geitleri*, indiv. Col., C4, Pebble 1, PCR_clone 2 | >12_ge_IS | KY704153 | 1809 |
| 22.10.2015 | *C. geitleri*, indiv. Col., C4, Pebble 1, PCR_clone 4 | >13_ge_IS | KY704154 | 1827 |
| 22.10.2015 | *C. geitleri*, indiv. Col., C4, Pebble 1, PCR_clone 7 | >14_ge_IS | KY704155 | 1827 |
| 22.10.2015 | *C. polonicus*, indiv col., C5, Pebble 3, PCR_clone 8 | >6_po_IS | KY704156 | 1831 |
| 22.10.2015 | *C. polonicus*, indiv col., C10, Pebble 6, PCR_clone 6 | >7_po_IS | KY704157 | 1831 |
| 22.10.2015 | *C. polonicus*, indiv col., C10, Pebble 6, PCR_clone 10 | >8_po_IS | KY704158 | 1833 |
| 22.10.2015 | *C. geitleri*, indiv. Col., C1, Pebble 3, PCR_clone 10_II | >15_ge_IS | KY704159 | 1802 |
| 22.10.2015 | *C. geitleri*, indiv. Col., C1, Pebble 3, PCR_clone 5_II | >16_ge_IS | KY704160 | 1801 |
| 22.10.2015 | *C. geitleri*, indiv. Col., C1, Pebble 3, PCR_clone 11_II | >17_ge_IS | KY704161 | 1803 |
| 22.10.2015 | *C. polonicus*, indiv col., C10, Pebble 6, PCR_clone 10_II | >10_po_IS | KY704162 | 1798 |
| 01.11.2014 | *C. geitleri* strain No. 1023 | >ge_1023 | KY704109 | 1803 |

Table S3: Cyanobacteria strain names and abbreviations used to construct the ML-phylogenetic tree shown in Fig. 6 (downloaded from Ribosomal Database Project (RDP), <https://rdp.cme.msu.edu/> , 22 March 2017 (Cole et al. 2014)

| **Taxonomic Name** | **Sequence accesss. No.** | **Abbreviation** | **Sequence length** |
| --- | --- | --- | --- |
| *Anabaena compacta* 118 | AJ293109 | >Anabcompac | 1033 |
| *Anabaena lemmermannii* 202A2 | AJ293104 | >Anablemmer | 1033 |
| *Anabaena planctonica* 71 | AJ293108 | >Anabplanct | 1033 |
| *Anabaena spiroides* PCC 9403 | AJ293116 | >Anabspiroi | 1033 |
| *Anabaenopsis* sp. 1A | AF516747 | >Anabaenops | 1033 |
| *Aphanizomenon* sp. 'BC-Aph 9601' | AJ245457 | >Aphanizome | 1034 |
| *Arthronema gygaxiana* UTCC 393 | AF218370 | >Arthrongyg | 1030 |
| *Arthrospira* sp. PCC 7345 | X75044 | >Arthrospir | 1032 |
| *Calothrix* sp. DCC D253 | X99213 | >Calothrix | 1028 |
| *C. geitleri*, indiv. Col. A6, Isar (PCR_clone11) | KY704139 | >1_ge_IS | 1033 |
| *C. geitleri*, indiv. Col. A6, Isar (PCR_clone17) | KY704140 | >2_ge_IS | 1033 |
| *C. geitleri*, indiv. Col. B6, Isar (PCR_clone1) | KY704141 | >3_ge_IS | 1033 |
| *C. geitleri*, indiv. Col. B6, Isar (PCR_clone7) | KY704144 | >6_ge_IS | 1032 |
| *C. geitleri*, indiv. Col., C1, Pebble 3, PCR_clone 10 | KY704150 | >9_ge_IS | 1030 |
| *C. geitleri*, indiv. Col., C1, Pebble 3, PCR_clone 11_II | KY704161 | >17_ge_IS | 1034 |
| *C. geitleri*, indiv. Col., C1, Pebble 3, PCR_clone 2 | KY704148 | >7_ge_IS | 1033 |
| *C. geitleri*, indiv. Col., C1, Pebble 3, PCR_clone 5_II | KY704160 | >16_ge_IS | 1032 |
| *C. geitleri*, indiv. Col., C1, Pebble 3, PCR_clone 9 | KY704149 | >8_ge_IS | 1033 |
| *C. geitleri*, indiv. Col., C2, Pebble 3, PCR_clone 5 | KY704151 | >10_ge_IS | 1034 |
| *C. geitleri*, indiv. Col., C2, Pebble 3, PCR_clone 6 | KY704152 | >11_ge_IS | 1033 |
| *C. geitleri*, indiv. Col., C4, Pebble 1, PCR_clone 2 | KY704153 | >12_ge_IS | 1015 |
| *C. geitleri*, indiv. Col., C4, Pebble 1, PCR_clone 4 | KY704154 | >13_ge_IS | 1033 |
| *C. polonicus*, indiv. Col. 28, Isar, u. Mikroskop isoliert | KY704124 | >1_po_IS | 1032 |
| *C. polonicus*, indiv col., C10, Pebble 6, PCR_clone 10 | KY704158 | >8_po_IS | 1035 |
| *C. polonicus*, indiv col., C10, Pebble 6, PCR_clone 10_II | KY704162 | >10_po_IS | 1038 |
| *C. polonicus*, indiv col., C10, Pebble 6, PCR_clone 6 | KY704157 | >7_po_IS | 1034 |
| *C. polonicus*, indiv col., C5, Pebble 3, PCR_clone 8 | KY704156 | >6_po_IS | 1033 |
| *C. polonicus*, indiv. Col. 16, Karwendelbach, v. Agar | KY704131 | >2_po_KW | 1031 |
| *C. polonicus*, indiv. Col. B9, Isar (PCR_clone14) | KY704145 | >3_po_IS | 1033 |
| *C. polonicus*, indiv. Col. B9, Isar (PCR_clone4) | KY704147 | >5_po_IS | 1034 |
| *C. polonicus*, indiv. Col. B9, Isar (PCR_clone2) | KY704146 | >4_po_IS | 1034 |
| *C. starmachii*, indiv. Col. D1, Nederbach(PCR_clone4) | KY704137 | >25_st_NB | 1033 |
| *C. starmachii*, indiv. colony B1, Nederbach(PCR_clone4) | KY704134 | >22_st_NB | 1032 |
| *C. starmachii*, indiv. Colony B1, Nederbach(PCR_clone9) | KY704135 | >23_st_NB | 1033 |
| *C. starmachii*, indiv. colony B1, Nederbach(PCR_clone1) | KY704132 | >20_st_NB | 1033 |
| *C. starmachii*, indiv. Col. 19, Nederbach, u. Mikroskop isoliert | KY704122 | >10_st_NB | 1033 |
| *C. starmachii*, indiv. Col. 25, Nederbach, u. Mikroskop isoliert | KY704123 | >13_st_NB | 1031 |
| *C. starmachii*, indiv. Col. 4, Fleck No. 1, Nederbach, u. Mikroskop isoliert | KY704125 | >14_st_NB | 1035 |
| *C. starmachii*, indiv. Col. 5, Fleck No. 1, Nederbach, u. Mikroskop isoliert, (PCR_clone B1) | KY704126 | >15_st_NB | 1035 |
| *C. starmachii*, indiv. Col. 5, Fleck No. 1, Nederbach, u. Mikroskop isoliert, (PCR_clone B9) | KY704127 | >16_st_NB | 1035 |
| *C. starmachii*, indiv. Col. 6, Fleck No. 2, Nederbach, u. Mikroskop isoliert | KY704128 | >17_st_NB | 1034 |
| *C. starmachii*, individ. Colony 11, Nederbach, v. Agar | KY704129 | >18_st_NB | 1034 |
| *C. starmachii*, individ. Colony 14, Nederbach, v. Agar | KY704130 | >19_st_NB | 1032 |
| *C. starmachii*, individ. Colony 2, Nederbach, v. Agar | KY704116 | >2_st_NB | 1036 |
| *C. starmachii*, individ. Colony 3, Nederbach, v. Agar | KY704117 | >3_st_NB | 1028 |
| *C. starmachii*, individ. Colony 4, Nederbach, v. Agar | KY704118 | >4_st_NB | 1040 |
| *C. starmachii*, individ. Colony 5, Nederbach, v. Agar | KY704119 | >5_st_NB | 1021 |
| *C. starmachii*, individ. Colony 6, Nederbach, v. Agar | KY704120 | >6_st_NB | 1036 |
| *C. starmachii*, individ. Colony 8, Nederbach, v. Agar | KY704121 | >Ch8_sp_ZB | 1029 |
| *C. geitleri* No1023 | KY704109 | >ge_1023 | 1033 |
| *C.* cf. *incrustans* | KY704115 | >incr_VR | 1029 |
| *C.* *investiens* UAM 386 | JQ070061 | >inv_UAM386 | 903 |
| *C. minutus* PCC 6605 | KY704112 | >6605_minut | 1033 |
| *C. polonicus* SAG 32.87 | KY704111 | >po_32_87 | 1034 |
| *C.* *polymorphus* Hindak 1984/5 | KY704110 | >pl_1984 | 1033 |
| *C.* sp. PCC 8308 | KY704114 | >sp_8308 | 1033 |
| *C. subglobosus* PCC 7430 | KY704113 | >sub_7430 | 1028 |
| *Chlorogloeopsis fritschii* PCC 6912 | AB093489 | >Chloroglo2 | 1033 |
| *Chlorogloeopsis* sp. Greenland_2 | DQ430997 | >Chloroglo4 | 1033 |
| *Chlorogloeopsis* sp. PCC 7518 | X68780 | >Chloroglo1 | 1032 |
| Chroococcales cyanobacterium LEGE 06123 | FJ589716 | >Chroococca | 1034 |
| *Chroococcidiopsis* sp. BB79.2 | AJ344552 | >Chroococc2 | 1034 |
| *Chroococcidiopsis* sp. BB96.1 | AJ344555 | >Chroococc3 | 1033 |
| *Chroococcidiopsis* sp. CC1 | DQ914863 | >Chroococc4 | 1034 |
| *Chroococcidiopsis* sp. CCMP1489 | AJ344556 | >Chroococci | 1033 |
| *Chroococcidiopsis* sp. LEGE 06174 | HQ832924 | >Chroococc6 | 1034 |
| *Chroococcidiopsis* sp. PCC 6712 | AB039004 | >Chroococc5 | 1032 |
| *Chroococcidiopsis thermalis* PCC 7203 | NC019695 | >ChrooTherm | 1034 |
| *Chroococcus* sp. VP2-07 | FR798925 | >Chroococcu | 1031 |
| *Chroogloeocystis siderophila* 5.2 s.c.1 | AY380791 | >Chroogloeo | 1034 |
| *Crinalium epipsammum* PCC 9333 | NC019753 | >Crinalepi | 1033 |
| *Cyanobium gracile* PCC 6307 | AF001477 | >Cyanobium | 1032 |
| *Cyanobium* sp. NS01 | AY172837 | >Cyanobium1 | 1032 |
| *Cyanobium* sp. PCC 7001 | AM709626 | >Synechoco9 | 1032 |
| *Cyanobium* sp. Y0011 | AY183114 | >Cyanobium2 | 1032 |
| *Cyanospira rippkae* PCC 9501 | AY038036 | >Cyanospira | 1033 |
| *Cyanothece* sp. 104 | DQ243687 | >Cyanothec2 | 1033 |
| *Cyanothece* sp. 115 | DQ243690 | >Cyanothec3 | 1033 |
| *Cyanothece* sp. ATCC 51142 | AF132771 | >Cyanothece | 1033 |
| *Cyanothece* sp. GSL007 | FJ546715 | >Cyanothec4 | 1033 |
| *Cyanothece* sp. PCC 7418 | AJ000708 | >Cyanothec1 | 1033 |
| *Dactylococcopsis salina* PCC 8305 | CP003944 | >Dactylococ | 1033 |
| *Dermocarpa* sp. MBIC10004 | AB058202 | >Dermocarpa | 1034 |
| *Dermocarpa* sp. MBIC10765 | AB058284 | >Dermocarp1 | 1034 |
| *Dermocarpella* sp. PCC 7326_SAG29.84 | AJ344559 | >Dermoincra | 1034 |
| *Escherichia coli* str. K-12 substr. MG1655 | U00096 | >Esch_coli | 1055 |
| *Euhalothece* sp. 'MPI 95AH10' | AJ000709 | >Euhalothec | 1033 |
| Euhalothece sp. 'MPI 96N304' | AJ000713 | >Euhalothe1 | 1033 |
| *Fischerella muscicola* PCC 7414 | AF132788 | >Fischmusci | 1031 |
| *Geitleribactron purpureum* Tovel-4 | KT819293 | >Geitlebac | 1014 |
| *Geitlerinema carotinosum* AICB 37 | AY423710 | >Geitlerin1 | 1034 |
| *Geitlerinema* sp. PCC 7105 | AB039010 | >Geitlerine | 1028 |
| *Gloeobacter violaceus* PCC 7421 | AF132790 | >Gloeoviola | 1031 |
| *Gloeocapsa* sp. KO30D1 | AB067579 | >Gloeocaps1 | 1032 |
| *Gloeocapsa* sp. PCC 73106 | AF132784 | >Gloeocapsa | 1035 |
| *Gloeocapsa* sp. PCC 7428 | NC019745 | >Gloeo7428 | 1034 |
| *Gloeothece* sp. PCC 6909/1 | EU499305 | >Gloeothece | 1033 |
| *Gloeotrichia echinulata* PYH6 | AM230703 | >Gloeotrich | 1033 |
| *Halospirulina* sp. 'CCC Baja-95 Cl.3' | Y18790 | >Halospir1 | 1033 |
| *Halospirulina* sp. 'MPI S3' | Y18789 | >Halospirul | 1034 |
| *Halospirulina tapeticola* (T) CCC Baja-95 Cl.2 | Y18791 | >Halospirta | 1033 |
| *Halothece* sp. PCyano42 | DQ058891 | >Halothece | 1032 |
| *Hormoscilla pringsheimii* SAG 1407-1 | KM019982 | >Hormopring | 1033 |
| *Komvophoron kgarii* strain Wanggoolba Creek | NR118555 | >Komvkgarii | 1022 |
| *Leptolyngbya angustata* UTCC 473 | AF218372 | >Leptoangus | 1032 |
| *Leptolyngbya boryana* PCC 73110 | X84810 | >Leptoabory | 1031 |
| *Leptolyngbya boryana* UTEX 'B 485' | AF132793 | >Leptoabor1 | 1032 |
| *Leptolyngbya foveolarum* Komárek 1964/112 | X84808 | >Leptooveol | 1032 |
| *Leptolyngbya* sp. VRUC 135 Albertano 1985/1 | X84809 | >Leptolyng1 | 1035 |
| *Leptolyngbya* sp. HBC2 | EU249128 | >Leptolyng3 | 1032 |
| *Leptolyngbya* sp. PCC 7104 | AB039012 | >Leptolyng2 | 1032 |
| *Leptolyngbya tenerrima* UTCC 77 | AF218368 | >Leptoatene | 1032 |
| *Limnothrix redekei* CCAP 1443/1 | AJ580007 | >Limnothred | 1030 |
| *Limnothrix redekei* NIVA-CYA 227/1 | AB045929 | >Limnotred1 | 1032 |
| LPP-group MBIC10012 | AB058209 | >LPPgroup | 1032 |
| LPP-group MBIC10086 | AB058224 | >LPPgroup1 | 1032 |
| *Lyngbya aestuarii* PCC 7419 | AJ000714 | >Lyngbyaaes | 1024 |
| *Microcoleus* sp. PCC 7113 | NC019738 | >Micro7113 | 1033 |
| *Microcystis aeruginosa* NIES 87 | D89031 | >Micraerugi | 1034 |
| *Microcystis aeruginosa* PCC 7806 | U03402 | >Micraerug4 | 1033 |
| *Microcystis aeruginosa* PCC 7941 | AJ133171 | >Micraerug3 | 1033 |
| *Microcystis aeruginosa* UWOCC S-15-b | AF139325 | >Micraerug1 | 1033 |
| *Microcystis elabens* NIES 42 | AB001724 | >Microelab | 1033 |
| *Microcystis flos-aquae* UWOCC N | AF139327 | >Micrflosaq | 1033 |
| *Microcystis holsatica* NIES 43 | D89036 | >Microhols | 1028 |
| *Microcystis* sp. CHAB731 | FJ595695 | >Microcysti | 1033 |
| *Microcystis viridis* NIES 102 | D89033 | >Micrviridi | 1031 |
| *Microcystis wesenbergii* NIES 111 | D89034 | >Micrwesenb | 1026 |
| *Myxosarcina* sp. PCC 7312 | AJ344561 | >Myxosarcin | 1034 |
| *Nodularia* sp. BCNOD9427 | AJ224447 | >Nodularia1 | 1034 |
| *Nodularia spumigena* PCC 73104 | AB039002 | >Nodulspumi | 1031 |
| *Okeania plumata* NAC8-45 | GU724196 | >Oscimargar | 1033 |
| *Okeania* sp. NAC8-18 | GU724195 | >Oscillator | 1033 |
| *Oscillatoria acuminata* PCC 6304 | AB039014 | >Osciacumin | 1025 |
| *Oscillatoria limnetica* MR1 | AJ007908 | >Oscilimnet | 1029 |
| *Oscillatoria miniata* NAC8-50 | GU724203 | >Osciaminia | 1032 |
| *Oscillatoria rosea* IAM M-220 | AB003164 | >Oscirosea | 1030 |
| *Oscillatoria sancta* PCC 7515 | AF132933 | >Oscisancta | 1025 |
| *Oscillatoria* sp. | AJ133106 | >Oscillato2 | 1032 |
| *Oscillatori*a sp. IAM M-117 | AB003163 | >Oscillato1 | 1030 |
| Oscillatoriales cyanobacterium OU_4 | GQ162220 | >Oscillato3 | 1031 |
| *Phormidium lumbricale* UTCC 476 | AF218375 | >Phormlumbr | 1033 |
| *Phormidium mucicola* IAM M-221 | AB003165 | >Phormimuci | 1029 |
| *Phormidium* sp. 195-A12 | EU282429 | >Phormidiu4 | 1032 |
| *Phormidium* sp. IAM M-99 | AB003169 | >Phormidiu3 | 1030 |
| *Phormidium* sp. MBIC10210 | AB183567 | >Phormidiu1 | 1034 |
| *Phormidium* sp. OU_10 | GQ162219 | >Phormidiu2 | 1032 |
| *Phormidium* sp. UTCC 487 | AF218376 | >Phormidium | 1033 |
| *Planktothrix agardhii* CYA 18 | X84811 | >Planktagar | 1032 |
| *Planktothrix agardhii* IAM M-244 | AB074507 | >Planktaga1 | 1033 |
| *Planktothrix rubescens* BC-Pla 9303 | AJ132251 | >Planktrub1 | 1033 |
| *Planktothrix rubescens* BC-Pla 9402 | AJ132250 | >Planktrube | 1033 |
| *Plectolyngbya hodgsonii* ANT.LG2.1 | AY493615 | >Pseudopho1 | 1032 |
| *Plectolyngbya hodgsonii* ANT.LPR2.2 | AY493583 | >Pseudophor | 1032 |
| *Plectonema* sp. F3 | AF091110 | >Plectonema | 1031 |
| *Pleurocapsa minor* SAG 4.99 | AJ344564 | >Pleuromino | 1033 |
| *Pleurocapsa* sp. PCC 7516 | X78681 | >Pleurocaps | 1033 |
| *Prochlorococcus marinus* subsp. null str. EQPAC1 | AF311217 | >Prochmarin | 1032 |
| *Prochlorococcus marinus* subsp. null str. TAK9803-2 | AF311220 | >Prochloro3 | 1032 |
| *Prochlorococcus marinus* subsp. pastoris str. NATL1 | AF311218 | >Prochloro4 | 1032 |
| *Prochlorococcus marinus* subsp. pastoris str. NATL2 | AF311219 | >Prochloro5 | 1032 |
| *Prochlorothrix hollandica* | AJ007907 | >Prochloro1 | 1032 |
| *Prochlorothrix hollandica* | AF132792 | >Prochloro | 1030 |
| *Pseudanabaena* sp. PCC 6802 | AB039016 | >Pseudanab1 | 1027 |
| *Pseudanabaena* sp. PCC 7367 | AB039018 | >Pseudanaba | 1033 |
| *Pseudanabaena* sp. PCC 7367 | AB039018 | >Pseudanab4 | 1033 |
| *Pseudanabaena* sp. PCC 7403 | AB039019 | >Pseudanab2 | 1028 |
| Pseudanabaenaceae cyanobacterium DPG1-KK5 | EF654067 | >Pseudanab3 | 1032 |
| *Raphidiopsis* sp. D9 ITS-L | EU552069 | >Raphidiops | 1032 |
| *Raphidiopsis* sp. D9 ITS-S | EU552070 | >Raphidiop1 | 1032 |
| *Spirulina* sp. CCC Snake P. Y-85 | Y18793 | >Spirulina3 | 1032 |
| *Spirulina* sp. MPIS4 | Y18792 | >Spirulina1 | 1034 |
| *Spirulina* sp. P7 | AF091109 | >Spirulina2 | 1034 |
| *Spirulina* sp. PCC 6313 | AM709631 | >Spirulina | 1029 |
| *Spirulina subsalsa* IAM M-223 | AB003166 | >Spirulsubs | 1032 |
| *Stanieria* sp. PCC 7301 | AB039009 | >Stanieria | 1036 |
| *Synechococcus* sp. HOG | AF448075 | >Synechoco6 | 1033 |
| *Synechococcus* sp. PCC 7002 | AJ000716 | >Synechoco1 | 1033 |
| *Synechococcus* sp. PCC 7003 | AB015059 | >Synechoco2 | 1033 |
| *Synechococcus* sp. PCC 73109 | AB015061 | >Synechococ | 1032 |
| *Synechococcus* sp. PCC 7502 | AF448080 | >Synechoco8 | 1030 |
| *Synechococcus* sp. PCC 8807 | AF448076 | >Synechoco7 | 1032 |
| *Synechococcus* sp. PCC7117 | AB015060 | >Synechoco3 | 1031 |
| *Synechococcus* sp. T71 | AF448073 | >Synechoco4 | 1034 |
| *Synechococcus* sp. UH7 | AF448074 | >Synechoco5 | 1033 |
| *Synechocystis* sp. PCC 6803 | BA000022 | >Synechocys | 1033 |
| *Trichodesmium contortum* | AF013028 | >Trichocont | 1032 |
| *Trichodesmium erythraeum* | AF013030 | >Trichoeryt | 1032 |
| *Trichodesmium* NIBB 1067 | X70767 | >Trichodesm | 1032 |
| *Trichodesmium tenue* | AF013029 | >Trichotenu | 1032 |
| *Trichodesmium thiebautii* | AF013027 | >Trichothie | 1032 |
| *Trichodesmium thiebautii* | AF091321 | >Trichothi1 | 1032 |
| Uncultured *C.* sp. clone 5f-17 | JF832304 | >C_5f_17 | 1033 |
| Uncultured *C.* sp. clone DB_BK_15_2 | KF417649 | >C_DB_BK_15 | 1001 |
| Uncultured *C.* sp. clone WB_5_9 | KF417650 | >C_WB_5_9 | 1001 |
| *Westiellopsis* sp. 985-1 | AJ544090 | >Westiellop | 1034 |
| *Xenococcus* sp. PCC 7307 | AB074510 | >Xenococcus | 1034 |

Table S4. Summary of 16S-ITS rDNA variability from *Chamaesiphon* strain cultures and isolated single colonies.

| ***Chamaesiphon* species** | **Length (bp)**  **min-med-max** | **Max. dissimilarity (%)** | **Average similarity (%)** | **N** |
| --- | --- | --- | --- | --- |
| *C. starmachii* | 608-642-667 | 26.7 | 9.4 | 24 |
| *C. polonicus* | 629-655-667 | 18.2 | 11.5 | 8 |
| *C. geitleri* | 638-661-664 | 13.2 | 7.0 | 17 |
| *Chamaesiphon* sp. (strains) | 610-640-681 | 25.8 | 16.3 | 7 |
| Total | 608-642-681 | 31.4 | 12.5 | 56 |

Table S5. Within *Chamaesiphon* morphospecies colony genetic variation as obtained from two or more PCR clones sequenced for 16S rDNA.

| Morphospecies | Colony | Date | n | Length (bp) | % dissimilarity (min - med - max) |
| --- | --- | --- | --- | --- | --- |
| *C. starmachii* | Fleck No. 1, Nederbach | 21.06.2010 | 2 | 1172 | 0.6 |
| *C. starmachii* | B1, Nederbach | 26.04.2011 | 4 | 1168 | 0 - 0.34 - 0.69 |
| *C. starmachii* | D1, Nederbach | 26.04.2011 | 3 | 1168 | 0 - 0.09 - 0.09 |
| *C. polonicus* | B9, Isar | 26.04.2011 | 3 | 1170 | 2.06 - 3.08 - 4.29 |
| *C. polonicus* | C10, Isar | 22.10.2015 | 3 | 1175 | 0 - 5.05 -5.05 |
| *C. geitleri* | A6, Isar | 26.04.2011 | 2 | 1167 | 0.0857 |
| *C. geitleri* | B6, Isar | 26.04.2011 | 4 | 1167 | 0 - 0.09 - 0.17 |
| *C. geitleri* | C1, Isar | 22.10.2015 | 6 | 1169 | 0.09 - 0.17 - 4.4 |
| *C. geitleri* | C2, Isar | 22.10.2015 | 2 | 1169 | 0.17 |
| *C. geitleri* | C4, Isar | 22.10.2015 | 3 | 1167 | 0.09 - 0.26 - 0.35 |

**References** (Tables S1, S3)

Cole, J. R., Q. Wang, J. A. Fish, B. Chai, D. M. McGarrell, Y. Sun, C. T. Brown, A. Porras-Alfaro, C. R. Kuske & J. M. Tiedje, 2014. Ribosomal Database Project: data and tools for high throughput rRNA analysis. Nucleic Acids Research 42(D1):D633-D642 doi:10.1093/nar/gkt1244.

Taton, A., S. Grubisic, E. Brambilla, R. De Wit & A. Wilmotte, 2003. Cyanobacterial diversity in natural and artificial microbial mats of Lake Fryxell (McMurdo Dry Valleys, Antarctica): a morphological and molecular approach. Appl Environ Microbiol 69(9):5157-5169.
